# Preparation, identification and molecular characterization of umami peptides from skipjack tuna meat: Insights from sensory evaluation and molecular dynamics simulations

**DOI:** 10.1016/j.fochx.2026.103595

**Published:** 2026-01-26

**Authors:** Fang-Fang Huang, Zhe Zhang, Yang-Yan Jin, Yu-Hui Zeng, Qi Zeng, Chang-Feng Chi, Bin Wang

**Affiliations:** a Zhejiang Provincial Engineering Technology Research Center of Marine Biomedical Products, School of Food and Pharmacy, Zhejiang Ocean University, Zhoushan 316022, China; b National and Provincial Joint Laboratory of Exploration and Utilization of Marine Aquatic Genetic Resources, National Engineering Research Center of Marine Facilities Aquaculture, School of Marine Science and Technology, Zhejiang Ocean University, Zhoushan 316022, China

**Keywords:** Skipjack tuna meat, Umami peptides, Molecular dynamics simulation, T1R1/T1R3 taste receptor, Sensory evaluation

## Abstract

In this study, novel umami peptides were identified from skipjack tuna protein hydrolysates using enzymatic hydrolysis, purification, sensory evaluation, and molecular modeling. A hydrolysate with a high degree of hydrolysis was prepared using papain and flavor protease, and the most umami-active fraction was obtained through ultrafiltration and chromatographic separation. Fourteen peptides were identified, among which HAHA, QEYGGG, YD, DFDNA, DVPAE, EADH, and EYF showed lower umami thresholds than monosodium glutamate. Molecular docking and 100 ns molecular dynamics simulations revealed that DFDNA and DVPAE displayed low RMSD values, while key residues in the T1R1 and T1R3 subunits remained structurally stable. High-affinity peptides formed persistent hydrogen bonds and π interactions with the receptor complex. Short peptide length and the presence of acidic and aromatic residues were crucial for receptor recognition. Overall, these results clarify the molecular basis of umami perception and provide practical guidance for designing natural peptide-based flavor enhancers for applications.

## Highlights

• Key umami peptides were identified and evaluated for taste potency.
• DFDNA, WYDY, and EYF showed strong umami activity with low taste thresholds.
• DFDNA and DVPAE formed stable complexes with the T1R1/T1R3 receptor.
• Acidic and aromatic residues were critical for peptide-receptor binding.

## 1 Introduction

Umami, recognized as the fifth basic taste alongside sweet, sour, salty, and bitter, stems from the Japanese word “umai” (delicious) and was first discovered by Japanese scientist Kikunae Ikeda in 1908. Despite its early identification, umami was not widely accepted as a fundamental taste until 2002, following significant advances in understanding its molecular and sensory mechanisms (Crowe-White et al., 2023). As a unique savory sensation mediated by specific taste receptors, umami primarily arises from glutamates, nucleotides, and small peptides, which interact synergistically with the umami taste receptor family, particularly the heterodimer T1R1/T1R3 and the metabolic glutamate receptor mGluR4(Jia et al., 2023; Zhang Sun-Waterhouse et al., 2019).

In recent years, umami peptides, which are small molecules typically ranging in size from 150 to 3000 Da, have emerged as novel umami substances of particular interest due to their ability to enhance food flavor and deliver rich, harmonious taste experiences (Jia et al., 2023; Zhao et al., 2019). Distinguished from other flavor compounds by their structural motifs, umami peptides often contain glutamic acid (Glu) or aspartic acid (Asp) residues critical to their savory properties (Wu et al., 2024). These peptides not only improve food palatability but also display remarkable stability during processing, making them highly versatile in food industry applications(Gao et al., 2024; Zhang et al., 2024). Beyond taste enhancement, umami peptides are increasingly recognized for their bioactive functions. Recent studies have demonstrated that food-derived peptides may contribute to antioxidant, antihypertensive, and antidiabetic activities, immune modulation, and metabolic health(Eze et al., 2025). Comprehensive reviews also highlight their potential in regulating gut microbiota, reducing inflammation, and supporting cognitive health(Eze et al., 2024). These findings underscore the dual role of peptides as both flavor enhancers and functional ingredients, aligning with current trends in developing foods with combined sensory and health benefits.

Marine organisms represent an abundant natural source of umami peptides, with fish (Zhao et al., 2023), mollusks (Li et al., 2020), and algae (Li et al., 2020) serving as key reservoirs. To date, more than 200 umami peptides have been identified from a wide range of sources. Concrete discoveries span various taxonomic groups. For instance, umami peptides have been successfully isolated from mollusks such as blue mussels (*Mytilus edulis*) and Pacific oysters (*Crassostrea gigas*)(Wang et al., 2024). Traditional fermented products like fish sauce have also yielded purified and characterized umami-active peptides(Fu et al., 2024). The conventional discovery of umami peptides from fish primarily relies on an activity-guided fractionation pipeline: enzymatic hydrolysis followed by sequential separation coupled with sensory **e**valuation, with final identification by LC-MS/MS(He et al., 2023). While foundational, this approach has key limitations. Sensory evaluation, though direct, suffers from subjectivity and low throughput. Conversely, in silico screening offers efficiency but its accuracy depends heavily on receptor model quality and existing data, risking false positives or missed novel structures(Sun et al., 2024). Overall, the process is often non-targeted and inefficient, struggling to directly link peptide sequences to complex sensory outcomes like pure umami versus bitter off-tastes. Therefore, developing integrated strategies that combine sensory guidance, in silico prediction, and advanced analytics is crucial for the more efficient(Wang et al., 2026) and rational discovery of novel, potent, and clean-tasting umami peptides from underutilized resources such as Skipjack tuna.

Among marine fish, tuna (*Thunnus)*, often referred to as “ocean gold,” stand out due to their high protein content, nutritional richness, and economic value(Jumilla-Lorenz et al., 2024). Tuna is renowned for its exceptional protein quality, providing essential amino acids, unsaturated fatty acids, vitamins, and minerals crucial for human health(Kar et al., 2024). The presence of high-value proteins in tuna muscle makes it an ideal candidate for the exploration and extraction of bioactive peptides, particularly those imparting umami flavor. Previous studies have identified umami peptides from diverse seafood species (Qi et al., 2022), however, the discovery and characterization of such peptides from tuna, alongside their structural mechanisms of taste perception, remain largely unexplored. We hypothesize that specific structural motifs (e.g., acidic/aromatic residues, short chain length) in tuna-derived peptides are critical for high-affinity, stable interaction with the human umami taste receptor (T1R1/T1R3). To test this, a multidisciplinary approach combining enzymatic hydrolysis, multisensory evaluation, virtual screening, and molecular simulation is employed. Umami-rich peptides are extracted through optimized enzymatic processes, purified, and identified via chromatography and mass spectrometry. Their taste properties are validated by sensory analyses and electronic tongue evaluation. Concurrently, computational simulations elucidate the peptide-receptor interactions. By integrating these experimental and theoretical analyses, this work seeks to advance the fundamental understanding of umami peptide-receptor mechanisms and structure-activity relationships. Ultimately, it aims to provide the food industry with potent natural flavor candidates and a molecular blueprint for developing clean-label, health-oriented food products, supporting strategies such as sodium reduction.

This study aims to decode novel umami peptides from Skipjack tuna (*Katsuwonus pelamis*) muscle using a multidisciplinary approach that combines enzymatic hydrolysis, multisensory evaluation, virtual screening, and molecular simulation. By optimizing enzymatic processes, umami-rich peptides are extracted, purified, and structurally identified through advanced chromatography and mass spectrometry techniques. Sensory analyses, incorporating electronic tongue evaluation and umami threshold determination, will validate the taste properties of these peptides. Simultaneously, virtual screening and molecular docking will elucidate the interactions between identified umami peptides and their receptors, providing mechanistic insights into peptide-receptor binding. By integrating multisensory techniques with computational simulations, this work provides novel insights into the structure-activity relationships and receptor interaction mechanisms of umami peptides. This study holds significant implications for the food industry, offering novel natural umami enhancers while contributing to the development of functional foods and bioactive peptides for human health applications.

## 2 Materials and methods

### 2.1 Materials and chemicals

Skipjack tuna meat was provided by Ningbo JinriFood Co., Ltd. (Zhoushan, China). Trypsin (product code: T8150，Tissue Culture Grade, activity: 250000.U./g) and alkaline protease (product code: B8360, BR grade, activity: ≥200,000 U/g) were obtained from Solarbio Science & Technology Co., Ltd. (Beijing, China), while flavor protease (product code: F888659，BR grade, activity: ≥200,000 U/g) and papain (product code: P6321，BR grade, activity: ≥200,000 U/g) were purchased from Ruiyong Biotechnology Co., Ltd. (Shanghai, China).

Citric acid and L-isoleucine were supplied by Qianwei Food Technology Co., Ltd. (Shanghai, China). Sucrose, sodium chloride, and monosodium glutamate were sourced from Xilong Scientific Co., Ltd. (Shanghai, China). DEAE-52 cellulose and Sephadex G-15 were procured from YuanYe Biotechnology Co., Ltd. (Shanghai, China). Citric acid and L-isoleucine were purchased from Qianwei Food Technology Co., Ltd. (Shanghai, China).

### 2.2 Protease screening via single-enzyme hydrolysis

Four proteases were employed for hydrolysis: trypsin (pH 8.0, 37 °C), flavor protease (pH 6.5, 50 °C), alkaline protease (pH 10.0, 45 °C), and papain (pH 6.0, 50 °C). Enzymatic hydrolysis was conducted with an enzyme dosage of 1% (*w*/w, relative to the substrate), a substrate-to-solvent ratio of 1:10, and a reaction time of 5 h. The optimal protease was selected based primarily on the umami intensity scores from sensory evaluation of the hydrolysates. Sensory evaluation is the most direct measure of our target attribute. The degree of hydrolysis (DH) was concurrently analyzed as a supportive indicator to monitor proteolytic efficiency and to provide context for the sensory results, as an extremely high or low DH can influence peptide profiles and taste quality. Enzymatic hydrolysis was performed under the optimal conditions for each enzyme, followed by inactivation at 100 °C for 10 min. The hydrolysate was centrifuged at 8000 rmp for 15 min, and the supernatant was collected, freeze-dried, and prepared into a 5 mg/mL solution for sensory evaluation.

### 2.3 Preparation of umami peptides from tuna meat via combined enzymatic hydrolysis

Hydrolysates produced by the two proteases demonstrated the strongest umami-enhancing properties, then flavor protease and papain were selected for use in a combined enzymatic hydrolysis process. Pretreated samples were first hydrolyzed using the endopeptidase papain under its optimal conditions (pH 6.0, 50 °C) with an enzyme dosage of 1% (*w*/w, relative to the substrate). After 2 h, the enzyme was inactivated by heating at 100 °C. The pH of the solution was then adjusted to 6.5 using 0.1 M hydrochloric acid or sodium hydroxide.

Subsequently, the exopeptidase flavor protease was applied for further hydrolysis at 50 °C for 3 h, using an enzyme dosage of 1%. Enzymatic activity was again terminated by heating at 100 °C. The total hydrolysis time was maintained at 5 h to align with the benchmark established during preliminary screening. The time allocation was optimized based on the distinct mechanisms of the two enzymes: a shorter initial papain hydrolysis (2 h) efficiently generated intermediate peptides, while a longer subsequent flavor protease hydrolysis (3 h) effectively refined these peptides to enhance umami intensity and reduce potential bitterness, leveraging their synergistic effect. The resulting hydrolysate was evaluated for umami flavor using sensory evaluation combined with electronic tongue analysis, and the composite hydrolysis product was designated as MF.

### 2.4 Determination of the degree of hydrolysis

The Degree of Hydrolysis (DH) was determined using the formaldehyde titration method to measure free amino nitrogen. Briefly, 5 mL of the hydrolysate was added to a beaker containing 60 mL of distilled water and stirred uniformly using a magnetic stirrer. The mixture was first titrated with a 0.1 M NaOH solution to pH 8.2. Then, 10 mL of 37% formaldehyde solution was added. After thorough mixing, the titration was continued with the 0.1 M NaOH solution until the pH reached 9.2. The volume of NaOH consumed to reach pH 9.2 was recorded as V1. A blank control was performed using 65 mL of distilled water following the same procedure, and the consumed NaOH volume was recorded as V2 [89]. The free amino nitrogen content was calculated as follows:

$$\text{Amino Nitrogen Content}\;\left(g/100\;\mathrm{mL}\right)=\left[\left(\text{V1}-\text{V2}\right)\times c\times 0.014\times 100\right]/V₀$$

where:c is the concentration of the NaOH standard titration solution (mol/L); 0.014 is the milliequivalent weight of nitrogen; 100 is the unit conversion factor; V₀ is the volume of the sample aliquot taken for analysis (mL).

The Degree of Hydrolysis (DH) was then calculated using the following formula:

DH (%) = (Amino Nitrogen Content in the Hydrolysate / Total Nitrogen Content in the Raw Material) × 100%.

### 2.5 Isolation and purification of umami peptides

#### 2.5.1 Ultrafiltration fractionation of umami peptides

The enzymatic hydrolysate was first filtered through a 0.22 μm aqueous phase membrane prior to ultrafiltration. Fractionation was performed using ultrafiltration membranes with molecular weight cut-offs (MWCOs) of 10, 5, and 1 kDa. Before use, the membranes were rinsed with 0.1 M NaOH solution for 15 min, followed by washing with ultrapure water until neutral pH was achieved. The ultrafiltration process was conducted under a frequency range of 35.1–35.6 Hz and a pressure range of 0.5–1.2 Pa. The resulting ultrafiltration fractions were designated as MF1 (MW > 10 kDa), MF2 (5 kDa < MW < 10 kDa), MF3 (1 kDa < MW < 5 kDa), and MF4 (MW < 1 kDa). Each fraction was freeze-dried and subsequently evaluated for umami intensity using sensory evaluation combined with electronic tongue analysis to identify the fraction with the strongest umami flavor.

#### 2.5.2 Fractionation of umami peptides via DEAE-52 anion exchange chromatography

DEAE-52 cellulose was used as the stationary phase, pre-swelled with ultrapure water according to the manufacturer's instructions before column packing. After equilibration with 2–3 column volumes of ultrapure water, the sample was loaded. The umami-intense fraction identified in Section 2.4.1 was prepared as a 150 mg/mL solution, filtered through a 0.22 μm microporous membrane, and 2 mL of the sample solution was applied to the column. Gradient elution was performed using ultrapure water, 0.05 M NaCl, and 0.1 M NaCl as eluents. Fractions were collected at a constant flow rate, with 15 mL collected per tube. Absorbance at 220 nm was monitored during the elution process. Fractions corresponding to the same elution gradient were pooled, concentrated, and subjected to desalination by dialysis in running water at room temperature for 24 h. The resulting samples were freeze-dried and evaluated for umami intensity. The fraction with the strongest umami flavor was selected for further purification.

#### 2.5.3 Fractionation of umami peptides using Sephadex G-15 gel filtration chromatography

The umami-intense fraction obtained from DEAE-52 anion exchange chromatography was prepared as a 30 mg/mL solution with ultrapure water, filtered through a 0.22 μm aqueous phase membrane, and subjected to further separation using a Sephadex G-15 column. Prior to column packing, the dry Sephadex G-15 gel medium was fully swollen in an excess of ultrapure water at room temperature for 24 h with intermittent stirring. After swelling, the gel slurry was de-aerated and packed into a glass chromatography column (1.6 cm × 100 cm) according to the manufacturer's instructions. The column was then equilibrated with at least 5 column volumes of the elution solvent before sample loading.

The separation was performed under the following conditions: a glass chromatography column (1.6 cm × 100 cm) was used, with a sample loading volume of 2 mL. Ultrapure water was used as the isocratic eluent. The flow rate was set at 1 mL/min, and fractions were collected every 4 min. After collection, the absorbance of each fraction was measured at 220 nm. Fractions corresponding to individual elution peaks were pooled, concentrated by evaporation, freeze-dried, and evaluated for umami intensity.

#### 2.5.4 Purification of umami peptides using high-performance liquid chromatography (HPLC)

The umami-active fraction exhibiting the highest taste intensity from Sephadex G-15 chromatography was dissolved in ultrapure water (1:10, *w*/*v*) and purified by RP-HPLC (Agilent 1260 Infinity II) using a ZORBAX SB-C18 column (5 μm, 4.6 × 250 mm) with 0.1% trifluoroacetic acid/water (A) and methanol (B) as mobile phases at 0.3 mL/min, where peaks detected at 214 nm and lyophilized for further analysis.

### 2.6 Amino acid sequencing and synthesis of umami peptides

The amino acid sequencing of the umami peptides was performed by Beijing Tailin Biotechnology Co., Ltd. (China). The peptide sequences were measured by a 494 Procise Protein Peptide Sequencer. Edman degradation was performed according to the standard program supplied by Applied Biosystems. Molecular masses of peptides were determined by a Q-TOF/MS with an ESI source. The identified peptides were synthesized by Shanghai Apeptide Co., Ltd. (China).

### 2.7 Sensory evaluation

Sensory evaluation of Zhejiang Ocean university does not require ethical permission from the Ethics Committee. All panelists participated voluntarily and provided their written informed consent before the study commenced. In addition, appropriate protocols were implemented to protect the rights, safety, and privacy of all participants throughout the research process. Personal information of the panelists was kept strictly confidential, and all data were anonymized prior to analysis to ensure privacy protection. Participants were informed of the study objectives, procedures, and their right to withdraw at any time without penalty. Sensory evaluation was conducted following the method described by huang et al. (Huang et al., 2025) with appropriate modifications. A sensory panel consisting of 10 trained laboratory members (5 males and 5 females, aged 22–30 years) was assembled. All panelists had undergone sensory training. The evaluations were performed in a dedicated sensory room under controlled environmental conditions. Freeze-dried samples from each separation step were dissolved in ultrapure water to a concentration of 1 mg/mL, filtered through a 0.22 μm membrane, and presented in a randomized order at room temperature. Samples were coded with three-digit random numbers. The evaluation procedure was as follows: Panelists were instructed to cleanse their palate with unsalted crackers and water between samples. For each sample, they were asked to take an adequate amount into their mouth, evaluate the immediate taste impression, and expectorate. They then rated the intensity of sour, sweet, bitter, salty, and umami tastes. The rating scale ranged from 0 to 10, where 0 indicated no taste and 10 indicated a prominent flavor. For the evaluation of sour, sweet, bitter, salty, and umami flavors, the following reference standards were used: citric acid (0.8 mg/mL), sucrose (10 mg/mL), L-isoleucine (2.5 mg/mL), sodium chloride (3.5 mg/mL), and monosodium glutamate (3.5 mg/mL), with a score of 5 representing the standard for each reference. The average score for each evaluation was calculated, and the sample with the highest umami intensity was selected for subsequent separation and purification.

### 2.8 Electronic tongue measurement

An electronic tongue taste analysis system (INSENT, SA402B, Japan) was used to measure the samples. Prior to data collection, the system underwent self-calibration to ensure the reliability and stability of the results. The test was performed at a temperature of approximately 25 °C. First, the membrane potential (Vr) was measured in the reference solution (0.3 mM tartaric acid and 30 mM KCl), followed by measurements in the sample solution (Vs). The taste sensor was then gently cleaned with the reference solution, and the potential (Vr0) in the reference solution was recorded after testing the sample. Sampling and cleaning were alternated, with each sample measured four times, and the first measurement was discarded (Zhang et al., 2023). The measurements were conducted by Hangzhou Yanqu Information Technology Co., Ltd. (China).

### 2.9 Prediction of umami peptide taste activity and physicochemical properties

The taste activity of identified umami peptides was predicted using the BIOPEP database (https://biochemia.uwm.edu.pl/biopep/start_biopep.php) to evaluate potential bioactivity and structure-sensory correlations, with bioactive fragment frequency (A) calculated as A = a/N (a: taste-active fragments; N: total residues). Additionally, peptide solubility was assessed using the Innovagen proteomics tool (http://www.innovagen.com/proteomics-tools), while toxicity was predicted via ToxinPred (https://webs.iiitd.edu.in/raghava/toxinpred/index.html) to ensure safety for potential food applications.

### 2.10 Descriptive evaluation of synthetic peptides

The umami threshold was analyzed using the Taste Dilution Analysis (TDA) method (Zhang et al., 2022). Synthetic peptides were prepared at a concentration of 1 mg/mL and serially diluted in a 1:1 (*v*/v) ratio. Panel members tasted the solutions in descending dilution order. A triangle test was used to determine the umami threshold. The threshold dilution factor (TD) was recorded when the taste difference between the diluted solution and two blank samples (water) was just detectable. The TD value was taken as the average of the panelists' responses, with a maximum error of one dilution level.

### 2.11 Umami-enhancing effect of synthetic peptides

The umami-enhancing threshold of synthetic peptides was determined using Comparative Taste Dilution Analysis (cTDA) (Huang et al., 2025), where peptides (1 mg/mL) were mixed with 0.3 mg/mL MSG solution and serially diluted (1:1, v/v). Panelists compared peptide-MSG mixtures against 0.3 mg/mL MSG control, with the last distinguishable dilution factor recorded as the threshold (averaged across panelists, ±1 dilution level). The umami enhancement factor (f) was calculated as f = C1/C2, where C1 is the control MSG concentration and C2 is the detectable MSG concentration in peptide-containing solutions.

### 2.12 Homology modeling of the umami receptor T1R1/T1R3

The amino acid sequences of T1R1 (Q7RTX1) and T1R3 (Q7RTX0) were obtained from the UniProtKB online database (https://www.uniprot.org/). The fish sweet taste receptor T1R2a-T1R3 (PDB ID: 5X2M) was used as the structural template for homology modeling. The 5X2M structure was imported into PyMOL and processed to remove water molecules and other ligands, retaining only chains a and b. The prepared template and T1R1/T1R3 sequences were then uploaded to the SWISS-MODEL platform (https://swissmodel.expasy.org/) using the “User Template” mode to generate individual homology models. The T1R1 and T1R3 VFD domains were subsequently assembled into a complete T1R1/T1R3 receptor model using the ZDock platform (https://zdock.umassmed.edu/). The reliability of the constructed model was validated using the Ramachandran plot analysis available on the SAVES v6.0 platform (https://saves.mbi.ucla.edu/). Potential ligand-binding pockets of the T1R1/T1R3 receptor were predicted using the DoGSiteScorer tool (https://proteins.plus/).

### 2.13 Molecular docking of umami peptides with the T1R1/T1R3 receptor

The peptide sequence was first converted to SMILES format using the online platform (https://www.novopro.cn/tools/peptide2smiles.html) and subsequently transformed into a 3D structural model using the corresponding tool (https://www.novopro.cn/tools/smiles2pdb.html). The resulting peptide structure was optimized in AutoDockTools-1.5.7 by adding hydrogen atoms, assigning charges, and optimizing geometry. Similarly, the T1R1/T1R3 receptor model was imported into AutoDockTools and prepared by adding hydrogen atoms, merging polar bonds, and assigning charges. The receptor binding pocket was specified, and molecular docking was performed using AutoDock Vina with the following grid box parameters: x = 55.245, y = 40.161, z = 4.424, and spacing = 0.375 Å. The resulting docking conformations were visualized using PyMOL 2.6.0 for 3D representations and Discovery Studio 2019 for 2D interaction diagrams.

### 2.14 Molecular dynamics simulations and binding analysis

Molecular dynamics (MD) simulations were conducted using GROMACS 2023 (AMBER ff99SB-ILDN) to characterize the structural basis of umami peptide recognition. After protein preparation and system solvation, each complex underwent standard minimization, equilibration，100 ns production runs were conducted. Trajectory analyses (RMSD, RMSF, SASA, RoG) were performed to evaluate structural stability and flexibility, while secondary-structure evolution was monitored using DSSP.

Binding energetics were assessed through MMPBSA calculations over the final 10 ns, including per-residue decomposition to identify key interface contributors. Computational alanine scanning further confirmed critical hot-spot residues responsible for stabilizing peptide–T1R3 interactions.

### 2.15 Statistical analysis

The data obtained from triplicate experiments are expressed as mean ± standard deviation (SD). Statistical analyses were performed using SPSS software (Version 26.0, Chicago, IL, USA). Differences among groups were evaluated using analysis of variance (ANOVA) followed by Duncan's multiple range test to determine statistical significance. Graphs and radar charts were generated using GraphPad Prism 8 and Origin 2021.

## 3 Results and discussion

### 3.1 Sensory evaluation and hydrolysis efficiency of tuna meat hydrolysates

**Fig. 1:**
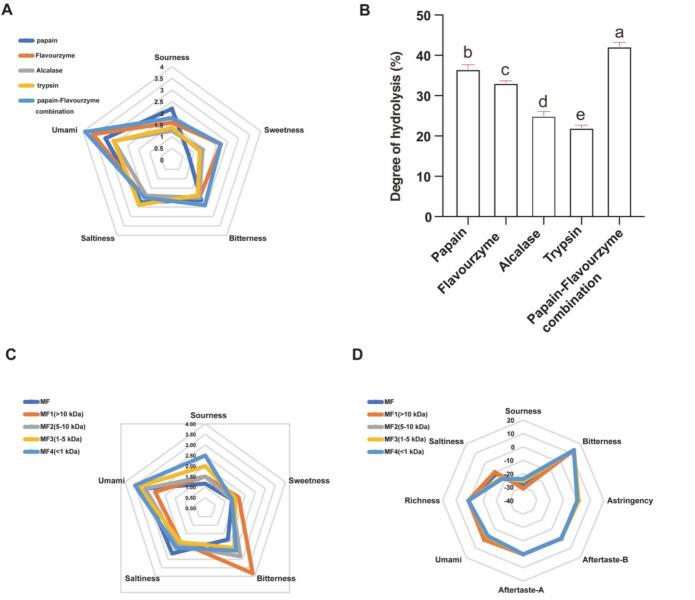
Compositional analysis of tuna meat hydrolysates and their sensory/electronic tongue evaluation. (A) Radar map of sensory evaluation profiles; (B) Degree of hydrolysis under different protease treatments;(C) Human sensory radar map; (D) Electronic tongue taste analysis relative to reference solution. Values sharing the same letter (a-e) are not significantly different (*P* > 0.05).

The enzymatic hydrolysis of tuna meat was systematically evaluated through both sensory analysis and DH measurements (Fig. 1A and B). Among single-enzyme treatments, flavor protease produced hydrolysates with the most pronounced umami taste, followed by papain, while papain produced hydrolysate achieved the highest DH (36.58 ± 1.11%) compared to that of flavor protease (33.14 ± 0.53%). Notably, the flavor protease hydrolysates demonstrated superior umami characteristics despite their slightly lower DH. Based on these findings, we employed a combined enzymatic approach using papain and flavor protease, which synergistically enhanced both hydrolysis efficiency and flavor profile. The combined treatment yielded a significantly higher DH (42.19 ± 1.05%, *P* < 0.05) and more intense umami taste compared to single-enzyme treatments, indicating more thorough protein breakdown and superior flavor development. This DH value is notably higher than those reported in many similar studies on tuna or other fish proteins. For instance, hydrolysis of yellowfin tuna dark meat with trypsin achieved an optimal DH of only about 19.89%(Liu et al., 2014), and hydrolysis of skipjack red meat with a protease complex resulted in a DH of 16.73% in a study focused on antioxidant activity (Abd El-Rady et al., 2023). Our significantly higher DH underscores the efficiency of the synergistic papain-flavor protease sequential system in degrading tuna myofibrillar proteins. Moreover, while protein hydrolysates from many fish species or by-products (e.g., herring, salmon) are often reported to develop intense bitterness or fishy off-flavors alongside umami(Abd El-Rady et al., 2023). Our hydrolysate (MF) was characterized by a dominant and clean umami taste as guided by sensory evaluation. This successful generation of a desirable flavor profile is directly attributed to our flavor-oriented screening strategy, which prioritized sensory evaluation over DH alone during protease selection. Our hydrolysate (MF) was characterized by a dominant and clean umami taste as guided by sensory evaluation. After sequential hydrolysis of tuna meat with papain (1% *w*/w, pH 6.0, 50 °C) for 2 h, followed by heat inactivation and pH adjustment to 6.5, and then with flavor protease (1% w/w, 50 °C) for 3 h prior to final heat inactivation, the resulting hydrolysate was selected for subsequent separation experiments.

This approach distinguishes our work from numerous studies that primarily aim to maximize DH for nutritional or antioxidant purposes. Our integrated process, from efficient hydrolysis to activity-guided fractionation, establishes a targeted pipeline for the discovery of novel umami peptides from Skipjack tuna.

### 3.2 Sensory and instrumental evaluation of ultrafiltration fractions

Ultrafiltration was employed for the preliminary fractionation of tuna muscle hydrolysates due to its operational simplicity, mild processing conditions, and ability to preserve peptide integrity without phase transitions or chemical modifications (Ai et al., 2024). As shown in Fig. 1C, sensory evaluation revealed that the MF4 fraction (MW < 1 kDa) exhibited significantly stronger umami intensity than both the unfractionated hydrolysate and other fractions (MF, MF1-MF3), consistent with previous reports that umami peptides are typically low-molecular-weight oligopeptides(Kong et al., 2019). To complement human sensory analysis, electronic tongue measurements were performed (Fig. 1D), which although not fully identical, showed a similar trend with MF4 displaying the highest umami response, followed by MF, MF3, MF2 and MF1. This instrumental analysis confirmed that umami intensity inversely correlated with molecular weight. The convergent results from both evaluation methods (sensory panel and electronic tongue) validated the selection of the MF4 fraction (<1 kDa) for subsequent purification via DEAE-52 ion-exchange chromatography.

### 3.3 Results of DEAE-52 anion-exchange chromatography

**Fig. 2:**
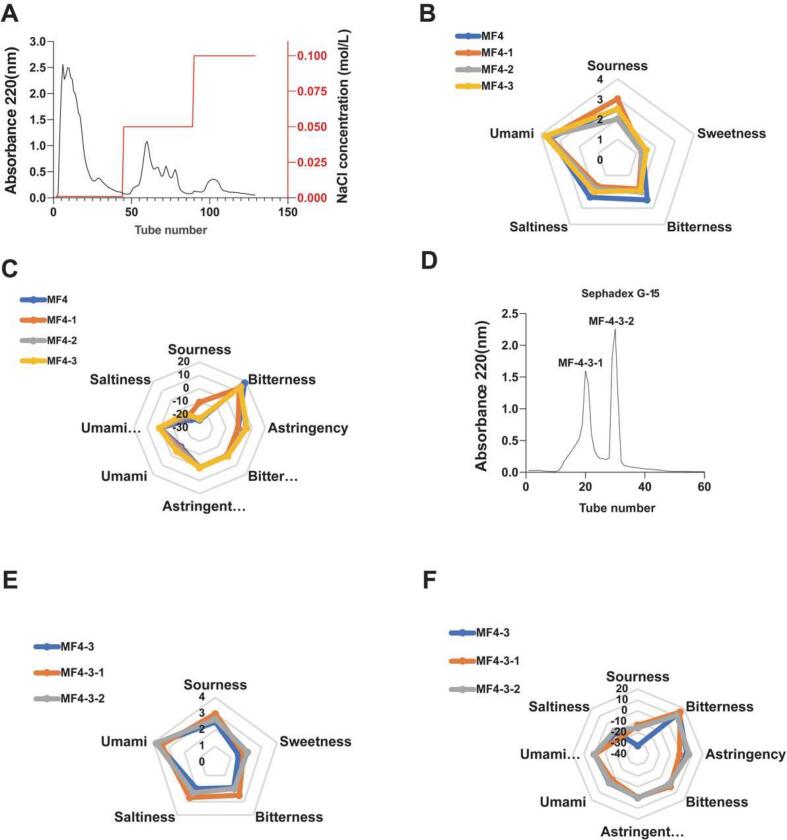
Sequential purification and sensory profiling of MF4-derived fractions from tuna meat hydrolysate. (A) Step-elution curve; (B) Sensory evaluation radar map; (C) Electronic tongue analysis relative to reference standard; (D) Elution curve of MF4–3; (E) Sensory evaluation radar map of collected fractions; (F) Electronic tongue analysis normalized to reference solution.

DEAE-52 ion-exchange chromatography is a technique that separates and purifies biomolecules based on the principle of ion exchange. As shown in Fig. 2A, the MF4 ultrafiltration fraction was prepared as a 150 mg/mL solution and eluted with NaCl solutions of varying concentrations. This process yielded three fractions: 0 M NaCl (MF4–1), 0.05 M NaCl (MF4–2), and 0.1 M NaCl (MF4–3). The sensory evaluation and electronic tongue analysis results of the desalted and freeze-dried samples are presented in Fig. 2B. The taste profiles of the three ion-exchange fractions were similar to those of the ultrafiltration sample (MF4) but exhibited enhanced overall taste intensities, particularly umami. Among the fractions, the umami intensity was highest in MF4–3, followed by MF4–2 and MF4–1 (Fig. 2C). Based on the combined results of sensory and electronic tongue evaluations, MF4–3 was selected for further purification in subsequent experiments.

### 3.4 Results of gel filtration chromatography

During gel filtration chromatography (using a Sephadex G-15 column), separation occurs based on molecular size. Larger molecules, which are excluded from the porous gel matrix, elute earlier in the void volume, while smaller molecules enter the pores and experience a longer path, resulting in delayed elution (Kim & Kim, 2022). As shown in Fig. 2D, two distinct peaks were observed after the fraction MF4–3 was separated on a Sephadex G-15 column, corresponding to fractions MF4–3-1 and MF4–3-2. Sensory evaluation and electronic tongue analysis results are presented in Fig. 2E and Table S1. Sensory analysis indicated that both MF4–3-1 and MF4–3-2 exhibited stronger umami intensities than the original MF4–3 sample, with MF4–3-2 showing the highest intensity, followed by MF4–3-1. However, the electronic tongue analysis (Fig. 2F, Table S2) provided slightly different results, ranking the umami intensities as MF4–3 > MF4–3-2 > MF4–3-1. This discrepancy in ranking between sensory and instrumental evaluations is not uncommon in taste science and primarily stems from their fundamentally different detection principles: human sensory evaluation directly reflects the specific biological activation of the umami receptor (T1R1/T1R3) and is considered the gold standard for perceived intensity, whereas the e-tongue detects non-specific, broad-spectrum electrochemical signals of the entire sample matrix(Ross, 2021). The complex matrix of the original MF4–3 fraction may contribute to a stronger composite e-tongue signal, while its purified subfractions (MF4–3-1 and MF4–3-2) demonstrated purer and stronger umami activity sensorily. Given the primary role of human sensory evaluation in identifying taste-active compounds, fraction MF4–3-2, which received the highest sensory score, was selected for further purification in subsequent experiments. The e-tongue data served as a valuable and objective complementary tool to monitor gross changes throughout the fractionation process.

### 3.5 HPLC separation and peptide sequence identification of fraction MF4–3-2

**Fig. 3:**
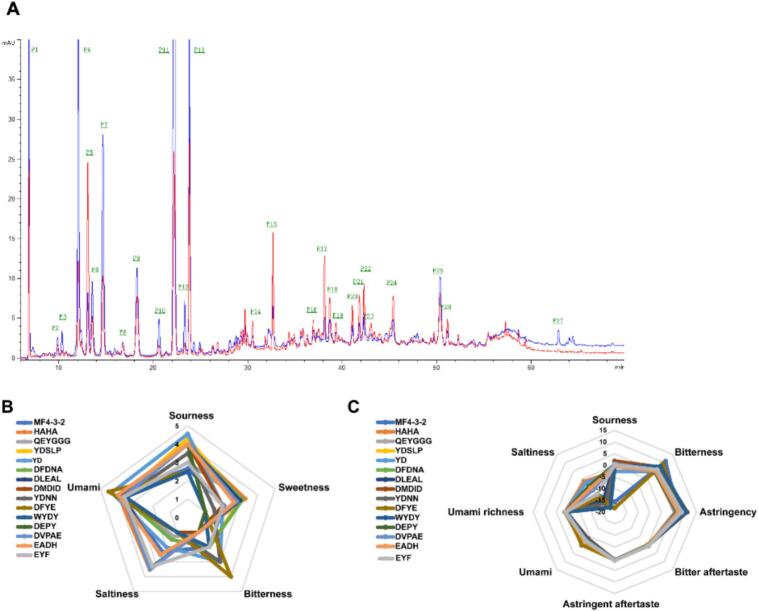
HPLC profile of MF4–3-2 tuna peptide and its umami taste characteristics. (A) HPLC profile of MF4–3-2 from tuna meat; (B) sensory evaluation radar of umami peptides; (C) taste radar of umami peptides based on reference solution.

As shown in Fig. 3A, the fraction MF4–3-2, which exhibited the strongest umami intensity after separation on Sephadex G-15 chromatography, was further purified using HPLC. This process resulted in the isolation of 27 subfractions. Subsequent sequencing analysis, excluding hydrophilic regions with no detectable signal peaks and peaks with low probability, identified 14 peptides from fraction MF4–3-2. These peptides were as follows: HAHA (P4, MW 434.44 Da), QEYGGG (P5, MW 609.58 Da), YDSLP (P6, MW 593.63 Da), YD (P7, MW 296.27), DFDNA (P9, MW 580.54 Da), DLEAL (P10, MW 559.60 Da), DMDID (P11, MW 607.63 Da), YDNN (P12, MW 524.48 Da), DFYE (P13, MW 572.56), WYDY (P15, MW 645.65 Da), DEPY (P17, MW 522.50 Da), DVPAE (P22, MW 529.54 Da), EADH (P24, MW 470.43 Da), EYF (P25, MW 457.47 Da). These identified peptides were selected as candidates for further synthesis and characterization to confirm their umami-enhancing properties.

### 3.6 Structural analysis, physicochemical characterization, and sensory evaluation of identified umami peptides

The frequency of taste-active fragments in the identified peptides was analyzed (Table 1). Among the 14 umami peptides, EADH exhibited the highest occurrence of umami-enhancing fragments (D, E, EA, and AD), followed by DEPY (D, E, and DE), while YDSLP showed the lowest frequency. Sweetness-active fragments were less prevalent, with DVPAE displaying the highest occurrence. Notably, DVPAE also contained the most sourness-active fragments (D, E, DV, and VP), whereas DLEAL and DVPAE showed the highest bitterness-active fragment frequency. Saltiness-active fragments were most abundant in DMDID.

**Table 1:** Predicted flavor presentation properties of amino acid fragments of peptide sequences.

Peptide sequence	Frequency of taste-active amino acid fragment sequences/active fragments				
	Umami	Sweetness	Sourness	Bitterness	Saltiness
HAHA	AH/0.25	A/0.50	AH/0.25	–	–
QEYGGG	E, EY/0.30	G/0.50	E/0.17	YG, YGG, EY/0.50	–
YDSLP	D/0.20	P/0.20	D, LP/0.40	P, L, SL/0.60	D/0.20
YD	D/0.50		D/0.50	–	D/0.50
DFDNA	D/0.40	A/0.20	D/0.40	F/0.20	D/0.40
DLEAL	D, E, EA/0.60	A/0.20	D, E/0.40	LE, L, DL, EA/0.80	D/0.20
DMDID	D/0.60	–	D/0.60	ID/0.20	D/0.60
YDNN	D/0.25	–	D/0.25	–	D/0.25
DFYE	D, E/0.50	–	D, E/0.50	F, FY/0.50	D/0.25
WYDY	D/0.25	–	D/0.25	W, DY/0.50	D/0.25
DEPY	D, E, DE/0.75	–	DE, D, E/0.75	P, PY/0.50	D, DE/0.50
DVPAE	D, E, AE/0.60	V, P, A/0.60	D, E, DV, VP/0.80	P, V, DV, PA/0.80	D/0.20
EADH	D, E, EA, AD/1.00	A/0.25	D, E/0.50	AD, EA/0.50	D/0.25
EYF	E, EY/0.67	–	E/0.33	F, YF, EY/1.00	–

Note: “-” indicates that the corresponding taste-active amino acid fragment within the peptide is not present in the database.

Key amino acids and dipeptides contributed to multiple taste modalities: Asp (D) and Glu (E) were associated with umami, sourness, and saltiness, while Glu-Ala (EA), Ala-Asp (AD), and Glu-Tyr (EY) were linked to both umami and bitterness. However, taste activity depends not only on fragment frequency but also on peptide length, composition, and sequence arrangement, necessitating sensory validation(Wang et al., 2024).

Physicochemical analysis (Table 2) revealed that all peptides were non-toxic (SVM scores <0). Most peptides had acidic pI values, except HAHA (near-neutral). While all peptides were predicted to be water-soluble, HAHA and WYDY showed poor solubility in silico, though experimental data confirmed HAHA's solubility, underscoring the need for empirical verification. These findings provide a foundation for understanding structure-taste relationships in umami peptides. Notably, WYDY exhibited the lowest docking energy (−10.4 kcal/mol) with the T1R1/T1R3 receptor, indicating the strongest predicted binding affinity. However, it did not yield the highest sensory score. This apparent discrepancy highlights that factors beyond static binding affinity critically influence perceived umami intensity. As suggested by its poor predicted solubility, WYDY's bioavailability at the taste receptor site may be limited. Furthermore, potent binding does not guarantee efficient receptor activation; the peptide's ability to induce the specific conformational change required for signal transduction is paramount. This observation reinforces that sensory validation is indispensable, as in silico docking, while a powerful screening tool, cannot fully capture the complex post-binding event (such as solubility, receptor activation dynamics, and oral stability) that ultimately determine taste perception. These integrated findings provide a more nuanced foundation for understanding structure-taste relationships in umami peptides.

**Table 2:** Prediction of physicochemical properties of peptide length, molecular weight, SVM score, pI, and solubility.

Peptide Sequence	Length	Molecular Weight	SVM Score	PI	Solubility
HAHA	4	434.44	−0.81	7.26	Poorly soluble
QEYGGG	6	609.58	−0.6	4	Highly soluble
YDSLP	5	593.63	−0.89	3.8	Highly soluble
YD	2	296.27	−0.79	3.8	Highly soluble
DFDNA	5	580.54	−0.93	3.57	Highly soluble
DLEAL	5	559.60	−0.93	3.67	Highly soluble
DMDID	5	607.63	−0.85	3.43	Highly soluble
YDNN	4	524.48	−0.76	3.8	Highly soluble
DFYE	4	572.56	−0.98	3.67	Poorly soluble
WYDY	4	645.65	−0.71	3.8	Poorly soluble
DEPY	4	522.50	−0.81	3.67	Highly soluble
DVPAE	5	529.54	−0.81	3.67	Highly soluble
EADH	4	470.43	−0.83	4.36	Highly soluble
EYF	3	457.47	−0.77	4.00	Highly soluble

### 3.7 Taste profile analysis of umami peptides from tuna meat

The sensory and electronic tongue evaluation results of 14 umami peptides from tuna meat are presented in Fig. 3B-C. A comparison of both evaluation methods indicates that the taste profiles of these peptides are similar to that of MF4–3-2. As shown in Fig. 3 B and 3C, the umami values of the 14 peptides do not vary significantly. The peptide DFYE exhibits the strongest umami taste, followed by YD, DLEAL, EADH, and DVPAE, which are slightly less intense. Compared to MF4–3-2, most identified peptides display a stronger sour taste. This could be due to the inherent sour characteristics of the peptides themselves or the incomplete removal of acidic amino acid residues during peptide synthesis, which may influence the taste of the solution. The enhanced astringency observed may be attributed to the residual presence of acetate salts and other chemical reagents used in the synthesis process.

### 3.8 Descriptive sensory evaluation of umami peptides from tuna meat

To objectively and accurately evaluate the umami intensity of umami peptides from tuna meat hydrolysate and comprehensively investigate their taste characteristics, this study employed descriptive sensory analysis, taste dilution analysis, and comparative taste dilution analysis to determine umami thresholds and umami enhancement thresholds, as well as to calculate the taste enhancement coefficients of the umami peptides.

As shown in Table 3, all identified peptides exhibited umami taste. The umami thresholds of DFDNA, DMDID, WYDY, DVPAE, EADH, and EYF were 0.125 mg/mL, while the thresholds for HAHA, QEYGGG, YDSLP, YD, DLEAL, YDNN, DFYE, and DEPY were 0.25 mg/mL-both of which are lower than that of monosodium glutamate (MSG) (0.3 mg/mL). This indicates that the 12 identified peptides possess stronger umami characteristics than MSG. Previous studies have demonstrated that umami peptides exhibit specific taste characteristics when acidic amino acids are located at the N-terminus and basic amino acids are at the C-terminus. Conversely, when basic amino acids are at the N-terminus and acidic amino acids are at the C-terminus, the peptides lack taste properties (Shiyan et al., 2021). The amino acid sequences of the umami peptides identified in this study align with these earlier findings. Among them, DFDNA, DMDID, DVPAE, EADH, and EYF exhibited particularly strong umami taste.

**Table 3:** Taste threshold, freshening threshold, taste enhancement coefficient and sensory description of umami peptides from tuna meat.

Peptide Sequence	Umami Threshold(mg/mL)	Umami Enhancement Threshold(mg/mL)	Taste Enhancement Coefficient	Sensory Description
HAHA	0.25	0.125	8	Umami, Sourness, Sweetness
QEYGGG	0.25	0.125	8	Umami, Sourness, Sweetness
YDSLP	0.25	0.25	4	Umami, Sourness, Sweetness
YD	0.25	0.125	8	Umami, Sourness, Slight Sweetness
DFDNA	0.125	0.125	8	Sourness, Umami, Slight Bitterness
DLEAL	0.25	0.5	2	Sourness, Umami, Slight Bitterness
DMDID	0.125	0.25	4	Umami, Pronounced Sourness
YDNN	0.25	0.25	4	Sourness, Umami, Bitterness
DFYE	0.25	0.5	2	Umami, Pronounced Bitterness
WYDY	0.125	0.25	4	Umami, Sourness
DEPY	0.25	0.25	4	Umami, Sourness
DVPAE	0.125	0.125	8	Umami, Sourness, Sweetness, and Astringency
EADH	0.125	0.125	8	Umami, Sourness, Sweetness
EYF	0.125	0.125	8	Umami, Sourness

Furthermore, hydrophobic amino acids have been shown to enhance the expression of umami taste in peptides. In this study, 11 synthesized peptides (HAHA, YDSL, DFDNA, DLEAL, DMDID, DFYE, WYDY, DEPY, DVPAE, EADH, EYF) were found to contain hydrophobic amino acids, such as alanine (A), leucine (L), phenylalanine (F), methionine (M), valine (V), proline (P), and asparagine (N). The comparative taste dilution analysis method is commonly used for the analysis of flavor enhancers. This method, developed based on taste dilution analysis, allows for both qualitative and quantitative analysis of flavor compounds in food. By comparing the taste differences between a 1 mg/mL umami peptide solution and a control MSG solution, the umami enhancement threshold of the 14 identified peptides was determined, and the flavor enhancement coefficient was calculated. As shown in Table 3, all peptides exhibited flavor enhancement abilities, with seven peptides including HAHA, QEYGGG, YD, DFDNA, DVPAE, EADH, and EYF having an enhancement threshold of 0.125 mg/mL. DLEAL and DFYE showed higher enhancement thresholds, at 0.5 mg/mL. The flavor enhancement coefficient more clearly demonstrated that peptides such as HAHA, QEYGGG, YD, DFDNA, DVPAE, EADH, and EYF had strong flavor-enhancing effects.

### 3.9 Homology modeling and structural validation of the umami receptor T1R1/T1R3

The heterodimeric T1R1/T1R3 receptor, particularly its Venus Flytrap domain (VFD), serves as the primary umami taste receptor by recognizing diverse amino acids. Since the crystal structure of human T1R1/T1R3 VFD remains unsolved, we generated a homology model using SWISS-MODEL (Fig. S1A). The template (5X2M) showed sequence similarities of 34.12% for T1R1 (left chain) and 38.18% for T1R3 (right chain), both exceeding the 30% threshold for reliable structure prediction. Ramachandran plot analysis (Fig. S1C) confirmed the model's robustness, with 100% of residues in allowed regions (89.7% favored, 9.9% allowed, 0.4% generally allowed), validating its stereochemical quality for molecular docking. Structural analysis revealed T1R1 adopts a closed conformation while T1R3 remains open, forming a ligand-binding cavity critical for umami perception. DoGSiteScorer-predicted binding pockets (Fig. S1B) comprised 88 residues with 122 hydrogen bond acceptors and 79 hydrophobic interactions, providing a structural basis for investigating umami peptide recognition mechanisms.

### 3.10 Molecular docking of umami receptors with umami peptides from tuna meat

**Fig. 4:**
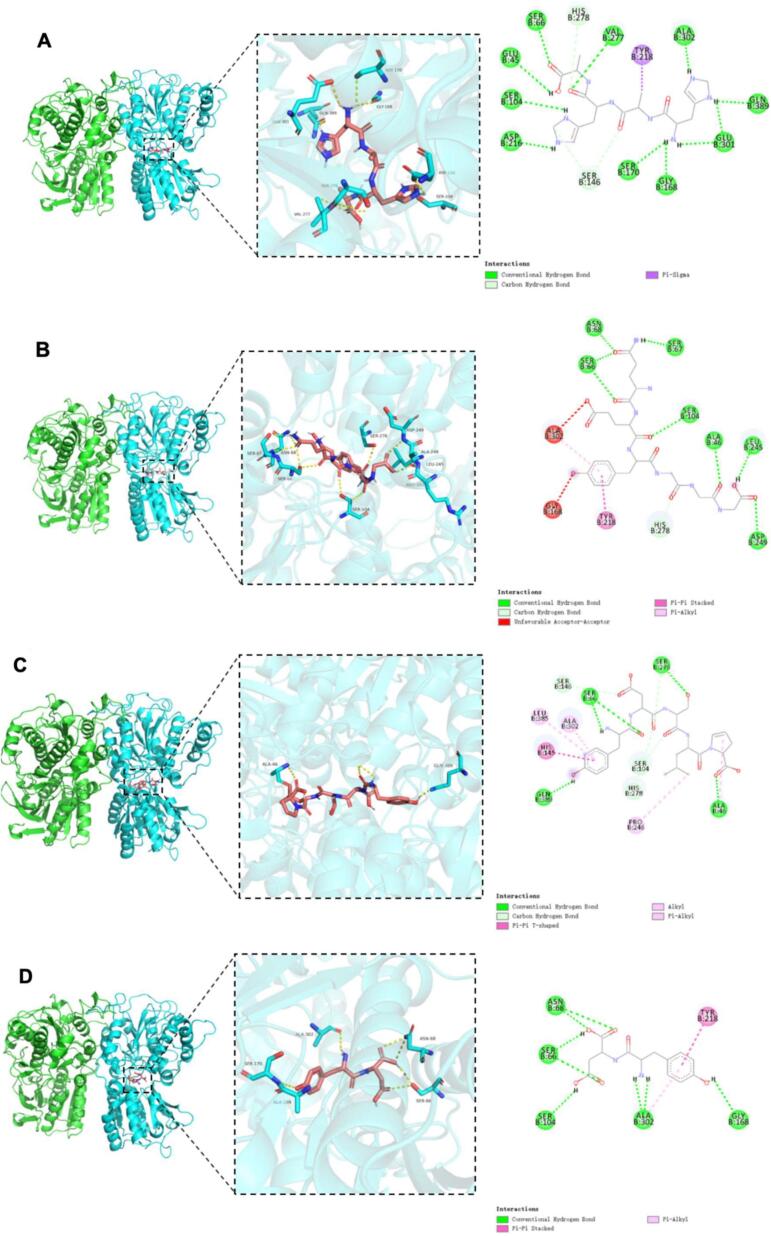

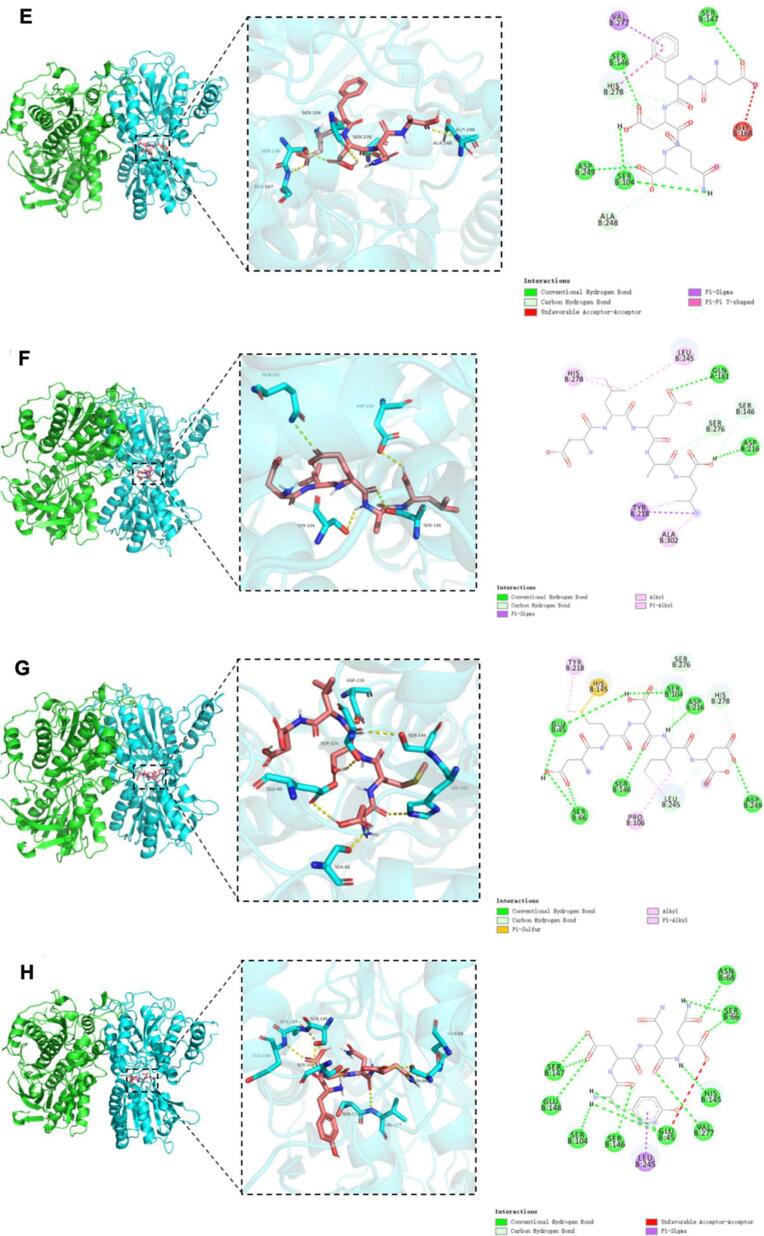

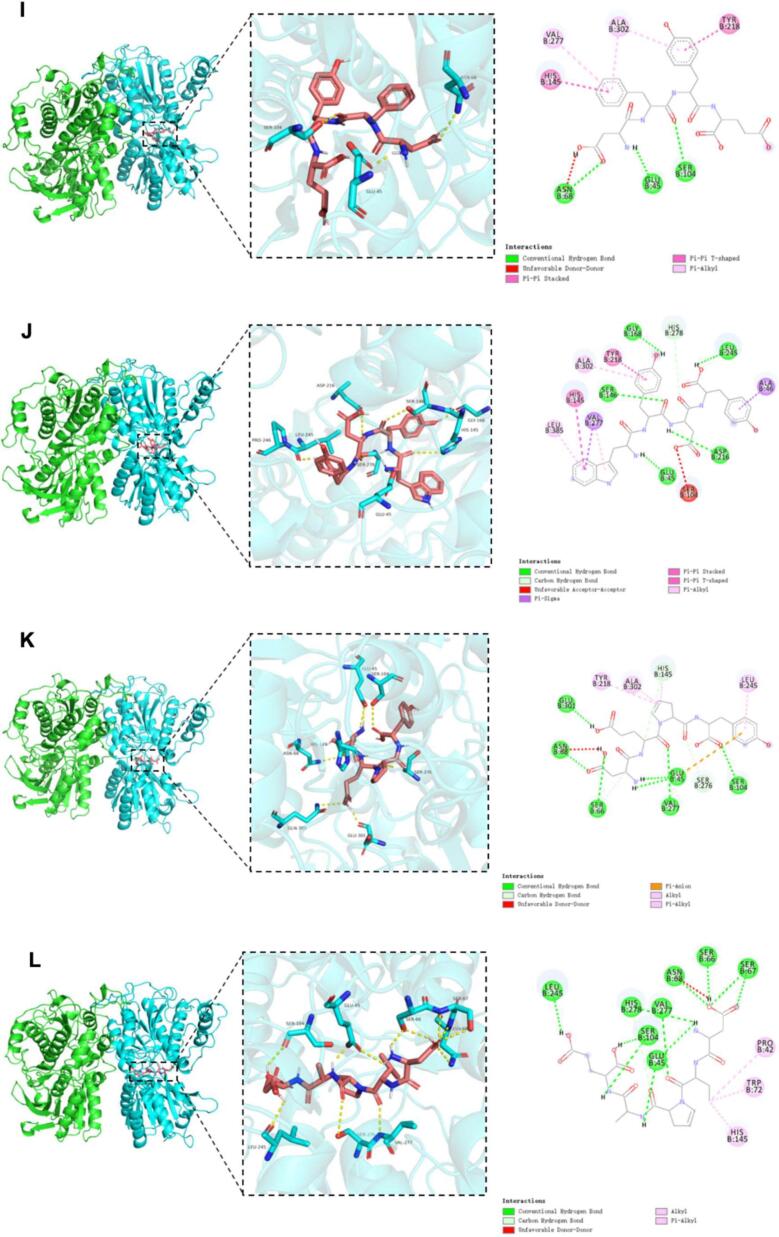

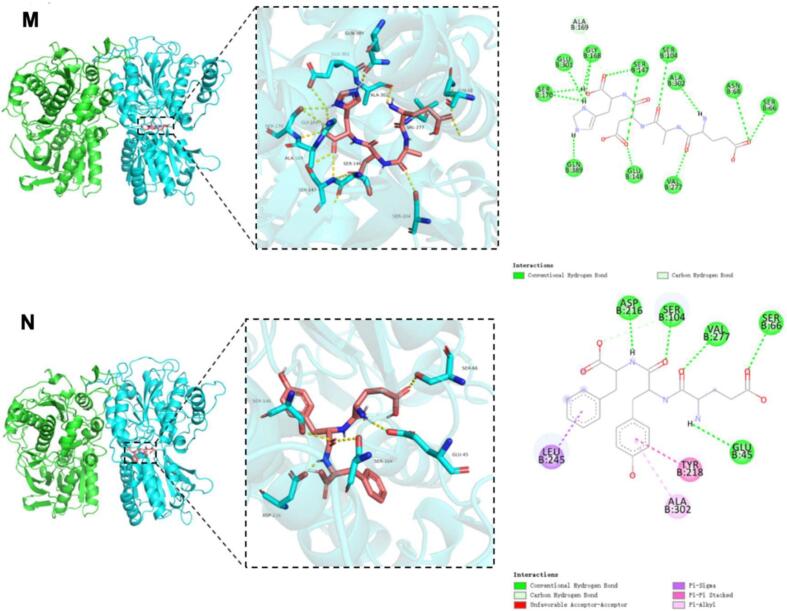
Molecular docking pose of 14 identified umami peptide with umami receptor T1R1/T1R3. Note: The peptides A-N represent the umami peptides HAHA, QEYGGG, YDSLP, YD, DFDNA, DLEAL, DMDID, YDNN, DFYE, WYDY, DEPY, DVPAE, and EADH, respectively, and their docking results with the active site of T1R3.

Fig. 4 illustrates the three-dimensional and two-dimensional binding models of 14 identified umami peptides docked with the T1R1/T1R3 receptor, showing their optimal docking conformations. As shown in Fig. 4, all 14 peptides bind to the receptor by embedding into the Venus flytrap domain (VFD) of the T1R3 subunit. Based on the binding affinity and binding modes analyzed from molecular docking results (Table S3), the peptides were ranked by docking energy from low to high as follows: WYDY, QEYGGG, DFYE, YDSLP, EYF, DFDNA = YDNN, EADH = HAHA, DEPY, DMDID, DVPAE, DLEAL, and YD. Among them, WYDY showed the lowest binding energy with T1R1/T1R3 at −10.4 kcal/mol, followed by QEYGGG (−9.6 kcal/mol) and DFYE (−9.4 kcal/mol).

However, no obvious correlation was observed between docking energy and peptide sequence length, which is consistent with previous findings(Dang et al., 2025). Consistent with sensory evaluations, the most potent umami peptides (DFDNA, DMDID, DVPAE, EADH, and EYF) demonstrated significantly lower binding energies than weakly active controls. However, discrepancies between docking energy rankings and sensory evaluation outcomes may result from structural deviations between the homology model and the actual crystal structure of the receptor. Further interaction analysis revealed that the binding of peptides to the T1R1/T1R3 receptor complex primarily involved hydrogen bonding and hydrophobic interactions (Table S3), consistent with previous studies (Zhang et al., 2023). As shown in Table S4, the 14 identified umami peptides can bind to 31 amino acid residues of the T1R3 subunit, with the key binding sites being Ser104, Ser66, Glu45, Ala302, His278, Val277, and Tyr218. Among these, Ser104 is the most frequent and forms hydrogen bonds with the peptides, followed by Ser66 and Glu45. This suggests that Ser104, Ser66, and Glu45 play a crucial role in the docking process between T1R3 and umami peptides. Chen et al. (Chen et al., 2022) found that the binding sites of T1R3 VFD primarily include Glu45, Ser104, Ser146, His145, Tyr218, and Val277, which stabilize the complex through hydrogen bonds or hydrophobic interactions. Similarly, Yang et al. (Yang et al., 2022) reported that the key active residues of T1R3 in their docking experiment with umami peptides include Ser276, His388, Val277, Ala302, Gln389, Ser146, Leu304, His145, Ser306, Ser104, His387, Thr305, Asn68, Glu301, and Arg54. Among these, Ser, Glu, and His contribute most to the interaction with the umami peptides. Our findings are consistent with these previous studies.

### 3.11 Molecular dynamics and energetic analysis reveal structural basis for umami peptide recognition by T1R3 receptor

**Fig. 5:**
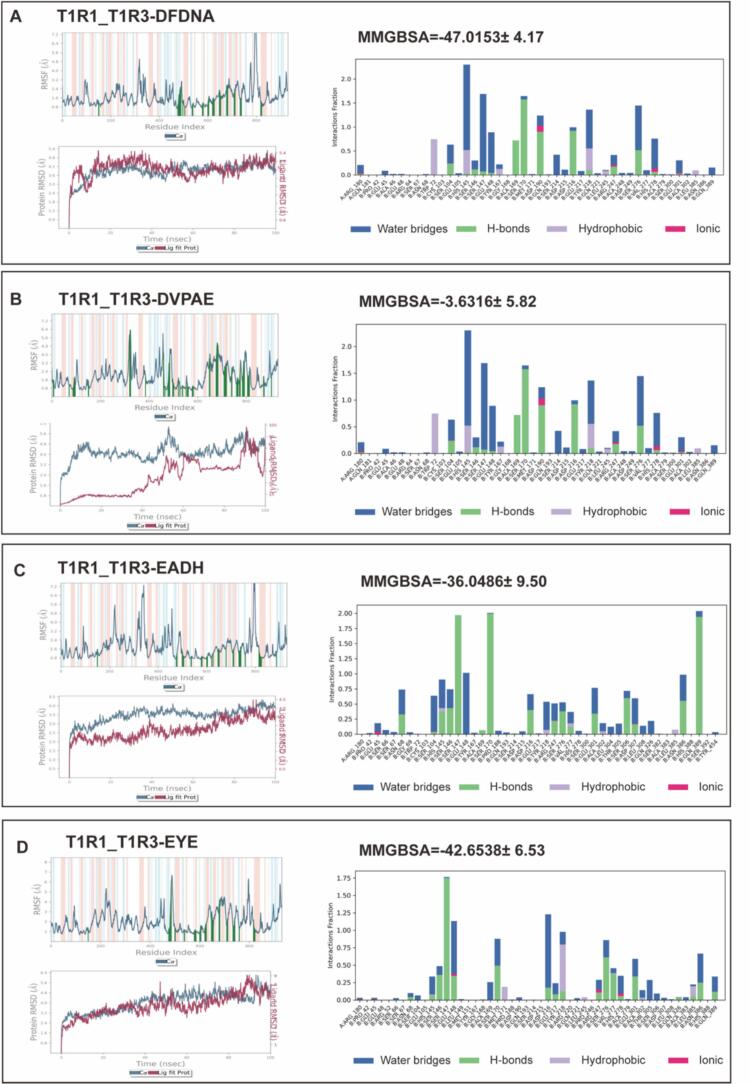

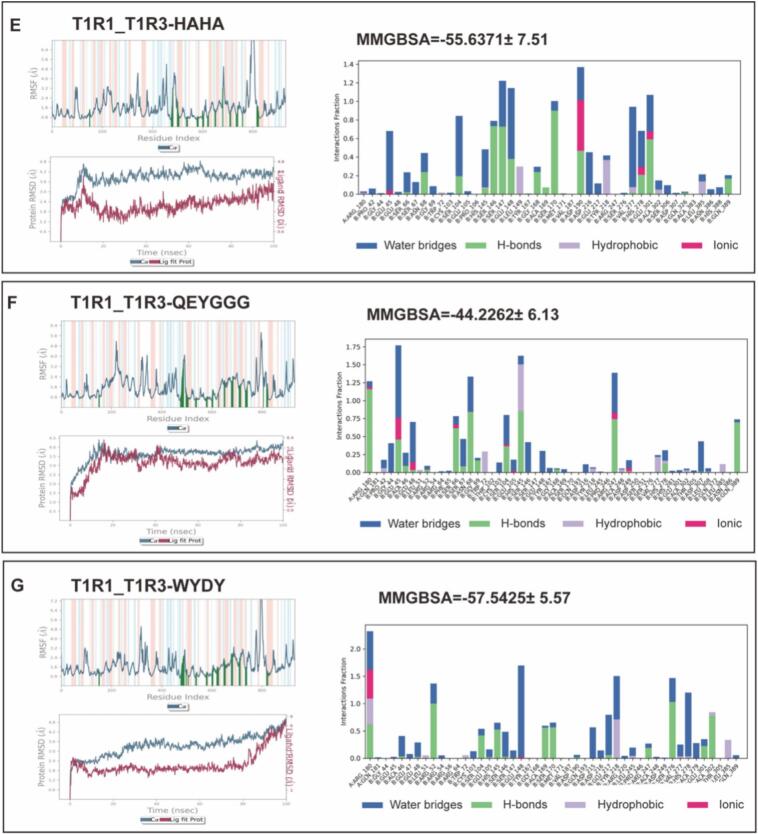
Molecular dynamics analyses. (A) DFDNA; (B) DVPAE; (C) EADH; (D) EYF; (E)HAHA; (F) QEYGGG; and (G) WYDY.

Based on their umami intensity and binding characteristics, seven key peptides (DFDNA, DVPAE, EADH, EYF, HAHA, QEYGGG, and WYDY) were selected for 100-ns molecular dynamics simulations with the T1R1/T1R3 receptor (Fig. 5). All systems equilibrated rapidly (∼10 ns). DFDNA and DVPAE exhibited the highest structural stability (average protein RMSD ∼2.0 Å), while HAHA and QEYGGG showed greater flexibility (RMSD ∼2.4–2.5 Å; ligand RMSD ∼3.0 Å). Crucially, key binding residues (e.g., T1R1-Ser382, T1R3-Ser104) remained highly stable across all complexes (RMSF <1.2 Å)(Fig. S2).

Simulations revealed distinct, stable interaction patterns. High-affinity peptides like WYDY and HAHA formed extensive hydrogen-bond networks with key receptor residues (e.g., Asp307, Glu301, Glu45), with occupancies exceeding 78%. These findings align with Feng et al.'s observation that collagen-derived peptides (GETGEAGER) interact strongly with T1R3's Arg291 and Glu296 through hydrogen bonding and electrostatic forces (Feng et al., 2024).

Comparative analysis underscores the superiority of our peptides. For instance, WYDY's strong π-cation interaction with Lys156 (−12.3 kcal/mol) exceeded the hydrophobic contacts reported for cod-derived peptides LVDKL (Zhao et al., 2024), correlating with its superior sensory threshold (0.03125 mmol/L). Similarly, the hydrophobic interaction profiles of QEYGGG and HAHA showed 20–30% higher occupancy than comparable cheese-derived peptides.

Notably, through comparative analysis of multiple umami peptide studies, we identified critical structural features governing receptor binding and taste perception. The presence of acidic residues (D/E) emerges as a fundamental characteristic, evidenced by the enhanced umami intensity of D/*E*-rich sequences such as Atlantic cod peptides LVDKL/ESKLI (Zhao et al., 2024) and Wuding chicken peptide HLEEEIK (Jia et al., 2024). High-affinity peptides typically exhibit a balanced combination of polar (D/E) and aromatic (F/W/Y) motifs, as observed in HAHA, GETGEAGER and various cheese and tuna-derived peptides. While 4–6 residue peptides demonstrate optimal binding characteristics, some longer sequences like GETGEAGERG (Feng et al., 2024) and EALEATAQ(Guo et al., 2024) maintain activity, though analysis of very long peptides (>8aa) requires more sophisticated modeling approaches. Notably, computational binding energy calculations (e.g., HAHA's − 55.64 kcal/mol vs DVPAE's − 3.63 kcal/mol) strongly correlate with sensory evaluation results and electronic tongue measurements, validating the predictive power of molecular dynamics simulations.

Our molecular dynamics simulations revealed distinct interaction patterns between high-affinity umami peptides (WYDY, HAHA) and the T1R1/T3 receptor, which align with the established paradigm that effective umami peptides rely on a combination of acidic and aromatic residues. For instance, WYDY formed stable hydrogen bonds with Asp307 and Glu301, while HAHA developed a four‑hydrogen-bond network via its C-terminal histidine, consistent with observations on collagen-derived peptides(Ding et al., 2019). To highlight the structural uniqueness of the Skipjack tuna-derived peptides identified in this study, a targeted comparison with representative marine-derived umami peptides is essential. Our findings reaffirm the critical role of acidic (Asp/Glu) and aromatic (Tyr/His) amino acid motifs within short peptide chains, a common feature observed in peptides derived from sources such as Atlantic cod(Moya Moreira et al., 2023) and cheese(Gu et al., 2024). In contrast, the peptides identified in our study exhibit distinct structural nuances, as exemplified by the pentapeptide DFNAA (Asp-Phe-Asp-Asn-Ala). It features two aspartic acid residues contiguously positioned in the central region of the pentapeptide, creating a concentrated, localized negative charge patch. This “-D-X-D-” pattern differs from the more commonly reported patterns where acidic residues are often singular, located at termini, or separated by multiple residues, suggesting a unique binding geometry for Skipjack tuna peptides.

This structural distinction translates into superior functional characteristics. Compared to the cod-derived peptide LVDKL(Zhao et al., 2024), our peptide WYDY demonstrates stronger electrostatic complementarity through a robust π-cation interaction with Lys156, which correlates with its significantly lower sensory threshold. Similarly, the hydrophobic clusters in QEYGGG and HAHA show higher interaction occupancy than analogous cheese-derived peptides(Gu et al., 2024), aligning with their enhanced umami intensity. These comparisons indicate that the novel peptides from Skipjack tuna achieve an optimized synergy of hydrogen bonding, electrostatic, and hydrophobic interactions, leading to greater binding stability and sensory potency. The computational binding energy calculations for peptides like HAHA strongly correlate with sensory and electronic tongue data, further validating the predictive power of our molecular simulations and the efficacy of these unique structural arrangements.

In summary, this study identifies novel umami peptides from Skipjack tuna characterized by unique structural patterns, such as central acidic clusters, which confer high receptor-binding stability and sensory potency. These findings provide the food industry with specific natural peptide candidates (e.g., EYF) as potent, clean-label alternatives to MSG. Furthermore, the elucidated structure-activity rules (acidic/aromatic residues, short chains) establish a rational design framework to guide efficient future R&D. Ultimately, these peptides serve as a practical tool for significant sodium reduction in savory foods, directly addressing key health and industrial reformulation challenges without compromising taste. However, a key limitation is the reliance on a homology-based receptor model for our computational simulations. While valuable for mechanistic hypothesis generation, this necessitates experimental validation. Future work should employ cellular functional assays and binding studies to confirm biological activity and mechanism. These steps are essential to translate the computational insights into the rational development of novel taste modulators.

## 4 Conclusion

This study establishes an effective pipeline for the discovery of novel umami peptides from Skipjack tuna. We identified 14 novel umami peptides and characterized the receptor binding mechanisms of seven high-affinity candidates (WYDY, QEYGGG, DFYE, DFDNA, DVPAE, EADH, EYF). Molecular dynamics simulations revealed that peptides such as DFDNA and DVPAE form exceptionally stable complexes (RMSD ∼2.0 Å) with the T1R1/T1R3 receptor, primarily through interactions with key residues (Ser104, Glu45, Ala302 in T1R3; Ser382, Glu338 in T1R1). Structural analysis further elucidated that a synergistic combination of acidic residues (Asp/Glu) for electrostatic interaction, aromatic side chains (Tyr/His) for π-stacking, and an optimal chain length of 4–6 residues constitutes a critical structural blueprint for potent umami recognition.These molecular insights are corroborated by sensory evaluation, where peptides like WYDY and QEYGGG exhibited strong umami-enhancing activity at low thresholds (0.125–0.25 mg/mL). Collectively, our work advances the fundamental understanding of umami peptide-receptor interactions. More importantly, it delivers a tangible toolkit for the food industry, comprising specific natural flavor candidates, a scalable enzymatic production process, and refined molecular design principles, paving the way for developing healthier, savory food products with reduced sodium.

## CRediT authorship contribution statement

**Fang-Fang Huang:** Validation, Investigation, Formal analysis, Data curation, Conceptualization. **Zhe Zhang:** Writing – original draft, Validation, Investigation, Formal analysis, Data curation, Conceptualization. **Yang-Yan Jin:** Validation, Methodology, Investigation, Formal analysis. **Yu-Hui Zeng:** Software, Methodology, Investigation. **Qi Zeng:** Writing – original draft, Supervision, Investigation. **Chang-Feng Chi:** Software, Methodology, Investigation. **Bin Wang:** Writing – review & editing, Supervision, Resources, Funding acquisition, Conceptualization.

## Declaration of competing interest

The authors declare no competing financial interests or personal relationships that could influence the work presented in this study.

## Data Availability

The data that has been used is confidential.

## Appendix A Supplementary data

Supplementary material Image 1
