# Supplementary material for: Preparation, identification and molecular characterization of umami peptides from skipjack tuna meat: Insights from sensory evaluation and molecular dynamics simulations
Source: Food Chem X. 2026 Jan 26;34:103595. doi: 10.1016/j.fochx.2026.103595 (PMC12874611; doi:10.1016/j.fochx.2026.103595)
Supplement: Supplementary file 1 — Supplementary material [file mmc1.docx]

**Preparation, identification and molecular characterization of umami peptides from Skipjack tuna meat: Insights from** **sensory evaluation and molecular dynamics simulations**

Fang-Fang Huang ^a,^ †, Zhe Zhang ^a,^ †, Yang-Yan Jin ^a^, Yu-Hui Zeng ^b^, Qi Zeng ^a,^ *, Chang-Feng Chi ^b^, Bin Wang ^a,^ *

*^a^ Zhejiang Provincial Engineering Technology Research Center of Marine Biomedical Products, School of Food and Pharmacy, Zhejiang Ocean University, Zhoushan 316022, China*

*^b^ National and Provincial Joint Laboratory of Exploration and Utilization of Marine Aquatic Genetic Resources, National Engineering Research Center of Marine Facilities Aquaculture, School of Marine Science and Technology, Zhejiang Ocean University, Zhoushan 316022, China*

† These authors have contributed equally to this work.

* Corresponding author:

Dr. Bin Wang,

Tel./Fax: +86-580-2554818; E-mail: wangbin@zjou.edu.cn, wangbin4159@hotmail.com.

Dr. Qi Zeng,

Tel./Fax: +86 580 2554818; E-mail address: 2022169@zjou.edu.cn

**Table S1**

Sensory evaluation radar map of collected fractions

|  | MF4-3 | MF4-3-1 | MF4-3-2 |
| --- | --- | --- | --- |
| Sourness | 2.5 | 3 | 2.67 |
| Sweetness | 1.5 | 1.83 | 2.08 |
| Bitterness | 1.92 | 2.5 | 2 |
| Saltiness | 2 | 2.67 | 2.33 |
| Umami | 3.75 | 3.5 | 3.83 |

**Table S2**

Electronic tongue analysis of collected fractions

|  | MF4-3 | MF4-3-1 | MF4-3-2 |
| --- | --- | --- | --- |
| Sourness | -32.1 | -13.18 | -15.61 |
| Bitterness | 13.76 | 15.27 | 10.85 |
| Astringency | 5.35 | -1.33 | 7 |
| Bitter aftertaste | 0.16 | 2.39 | 0.37 |
| Astringent aftertaste | 0.21 | -0.06 | 0.42 |
| Umami | -2.77 | -6.47 | -3.41 |
| Umami richness | 0.32 | 0.08 | 0.56 |
| Saltiness | -11.79 | -18.02 | -9.47 |

**Table S3**

Molecular docking binding energy and functional bonds of 14 identified umami peptide from tuna meat

| Peptide Attribute | Affinity (kcal/mol) | Hydrogen Bonds | Hydrophobic | Electrostatic | Miscellaneous |
| --- | --- | --- | --- | --- | --- |
| HAHA | -8.4 | 14 | 1 | - | - |
| QEYGGG | -9.6 | 9 | 2 | - | - |
| YDSLP | -9.2 | 9 | 5 | - | - |
| YD | -7.6 | 8 | 2 | - | - |
| DFDNA | -8.8 | 8 | 2 | - | - |
| DLEAL | -7.7 | 5 | 6 | - | - |
| DMDID | -8.0 | 14 | 3 | - | 1 |
| YDNN | -8.8 | 13 | 1 | - | - |
| DFYE | -9.4 | 3 | 5 | - | - |
| WYDY | -10.4 | 6 | 7 | - | - |
| DEPY | -8.2 | 10 | 4 | 1 | - |
| DVPAE | -7.9 | 13 | 3 | - | - |
| EADH | -8.4 | 15 | - | - | - |
| EYF | -8.9 | 6 | 3 | - | - |

Note: "-" indicates the absence of relevant functional bonds.

**Table S4**

Docking sites and interaction forces between umami peptides and T1R1/T1R3

|  | HA-4 | QG-6 | YP-5 | YD-2 | DA-5 | DL-5 | DD-5 | YN-4 | DE-4 | WY-4 | DY-4 | DE-5 | EH-4 | EF-  3 |
| --- | --- | --- | --- | --- | --- | --- | --- | --- | --- | --- | --- | --- | --- | --- |
| HIS278 | + | + | + |  | +++ | ++ | ++ |  |  | + |  | ++ |  |  |
| SER67 |  | + |  |  |  |  |  |  |  |  |  | ++ |  |  |
| VAL277 | + |  |  |  | + |  |  | + | + | ++ | + | + | + | + |
| SER276 |  |  | ++ |  |  | ++ | + |  |  |  | + |  |  |  |
| TYR218 | + | + |  | + |  | ++ | + |  | + | + | + |  |  | + |
| ALA302 | + | ++ | + | +++ |  | + |  |  | ++ | + | + |  | + | + |
| LEU245 |  | + |  |  |  | + | ++ | + |  | + | + | + |  | + |
| ASP249 |  | + |  |  | + |  | + |  |  |  |  |  |  |  |
| GLN389 | + |  | + |  |  |  |  |  |  |  |  |  | + |  |
| GLU301 | ++ |  |  |  |  |  |  |  |  |  | + |  | + |  |
| GLY168 | + | + |  | + | + |  |  |  |  |  |  |  | ++ |  |
| SER170 | + |  |  |  |  |  |  |  |  |  |  |  | ++ |  |
| SER146 | ++ |  | + |  | + | + | + | ++ |  | + |  |  |  |  |
| ASP216 | + |  |  |  |  | + | + |  |  | + |  |  |  | + |
| SER104 | + | + | ++ | + | ++ |  | +++ | + | + | + | + | ++ | + | ++ |
| ASN68 |  | + |  | ++ |  |  |  | + | + |  | ++ | + | + |  |
| ALA169 |  |  |  |  |  |  |  |  |  |  |  |  | + |  |
| GLU45 | + |  |  |  |  |  | ++ | +++ | + | + | +++ | ++ |  | + |
| SER66 | + | ++ | ++ | ++ |  |  | ++ | ++ |  |  | ++ | + | + | + |
| ALA46 |  | + | ++ |  |  |  |  |  |  | + |  |  |  |  |
| PRO246 |  |  | + |  |  |  |  |  |  |  |  |  |  |  |
| HIS145 |  |  | + |  |  |  | + | + | + | + | ++ | + |  |  |
| LEU385 |  |  | + |  |  |  |  |  |  | + |  |  |  |  |
| SER147 |  |  |  |  | + |  |  | ++ |  |  |  |  | ++ |  |
| ALA248 |  |  |  |  | + |  |  |  |  |  |  |  |  |  |
| GLN181 |  |  |  |  |  | + |  |  |  |  |  |  |  |  |
| PRO106 |  |  |  |  |  |  | + |  |  |  |  |  |  |  |
| GLU148 |  |  |  |  |  |  |  | + |  |  |  |  | + |  |
| GLY168 |  |  |  |  |  |  |  |  |  | + |  |  |  |  |
| PRO42 |  |  |  |  |  |  |  |  |  |  |  | + |  |  |
| TRP72 |  |  |  |  |  |  |  |  |  |  |  | + |  |  |

Note: The "+" symbol indicates the number of interactions between the ligand and the receptor.


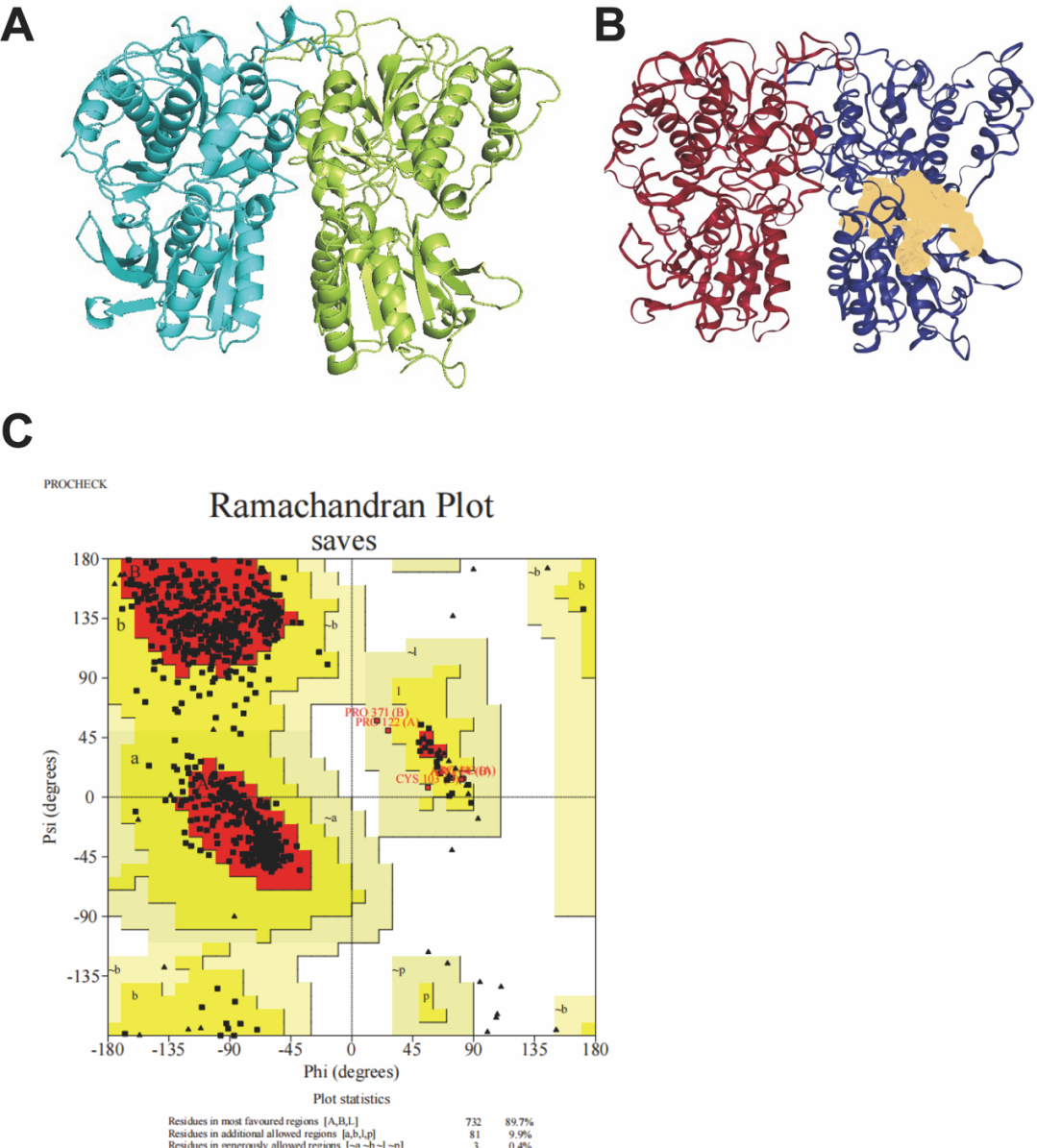


**Fig. S1. Computational modeling of umami receptor T1R1/T1R3 venus flytrap domain (VFD). (A) Homology model structure; (B) Predicted ligand-binding pocket surface; (C) Ramachandran plot validation.**


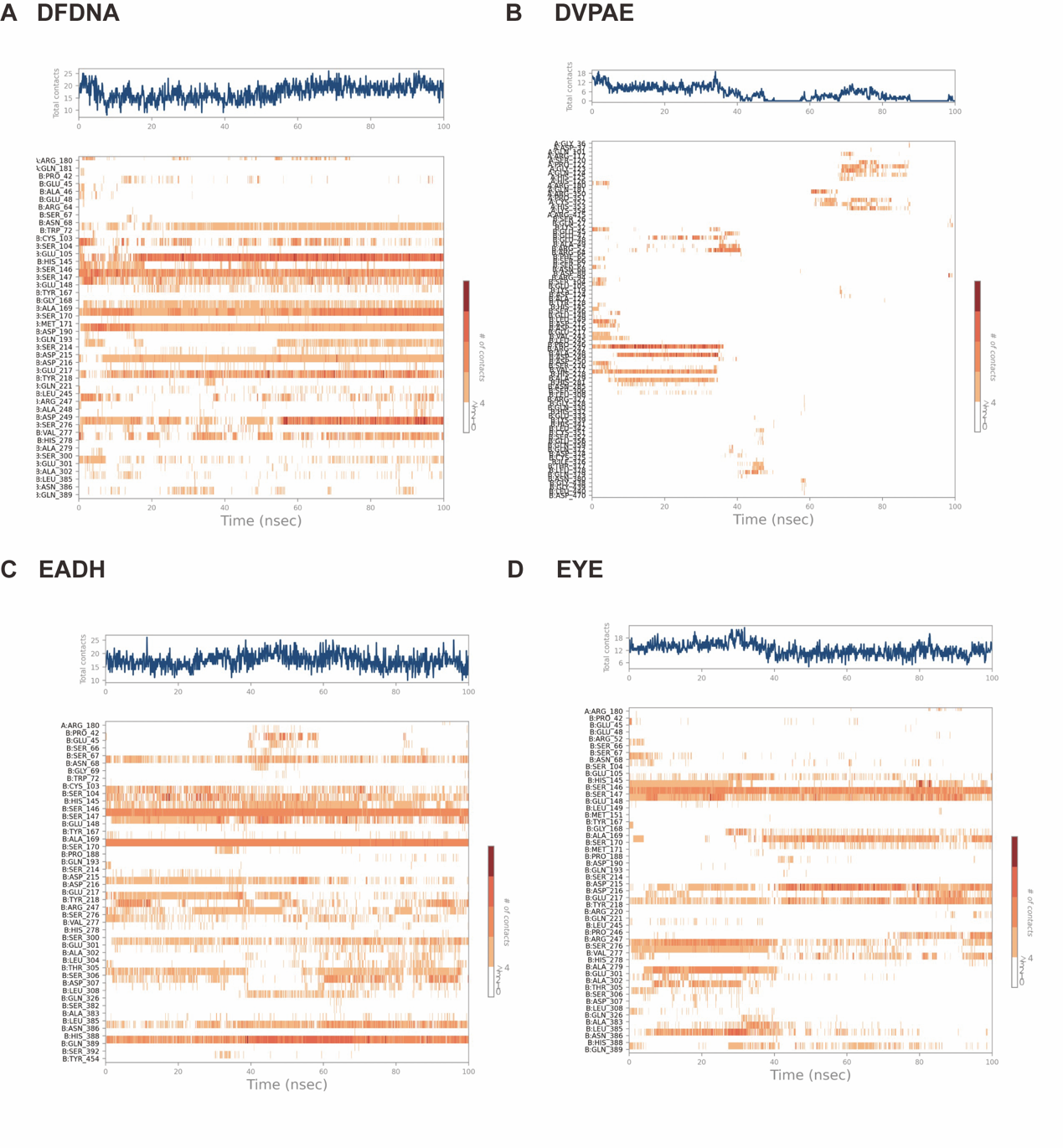

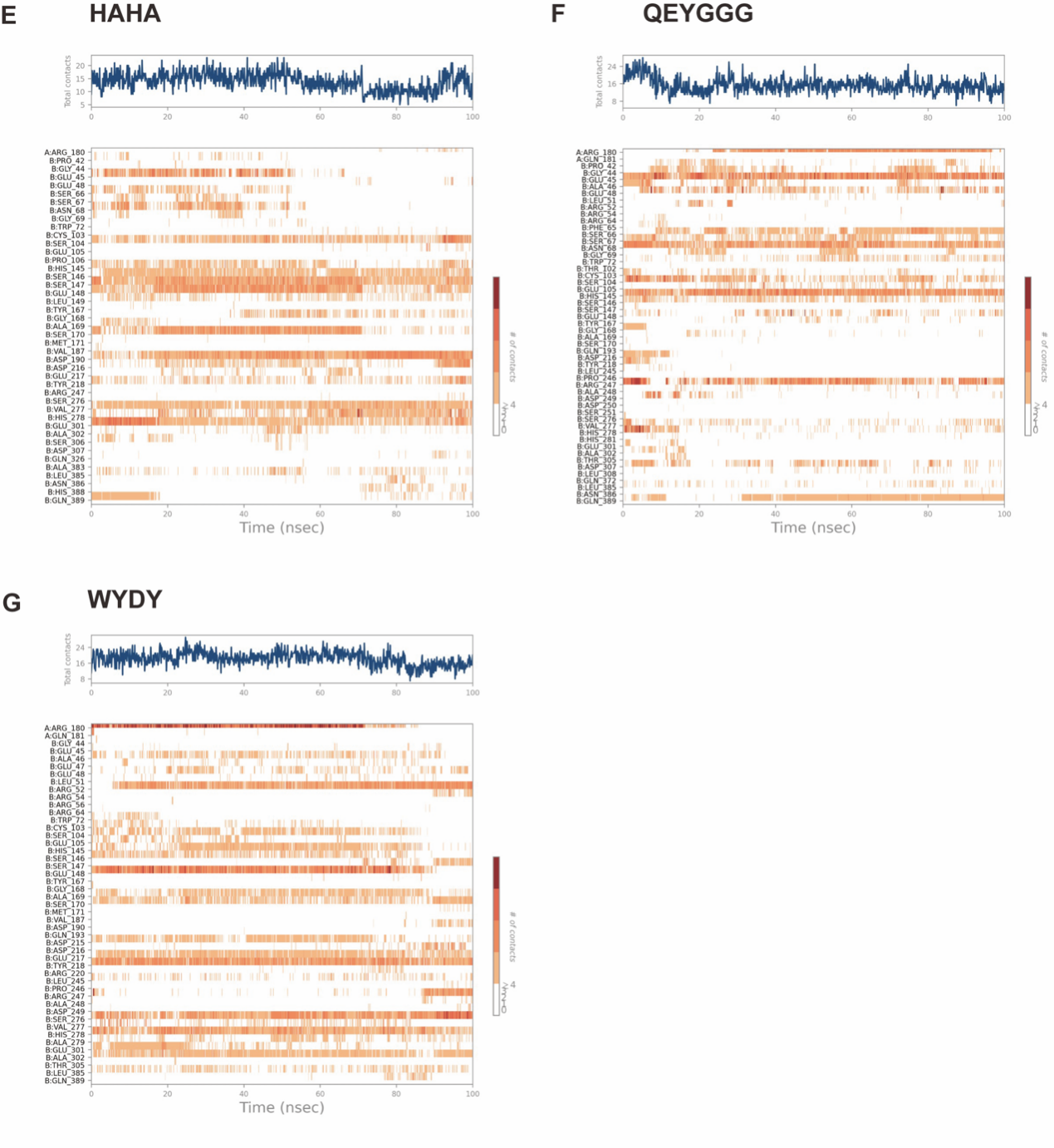


**Fig. S2. A timeline representation of the interactions and contacts (H-bonds, Hydrophobic, Ionic, Water bridges). (A) DFDNA; (B) DVPAE; (C) EADH; (D) EYF; (E)HAHA; (F) QEYGGG; and (G) WYDY.**
